# P036 Peeling back the layers: Takayasu’s arteritis mimicking isolated carotid artery dissection

**DOI:** 10.1093/rap/rkag113.066

**Published:** 2026-11-03

**Authors:** Rehab Mohammed, Anu Desai

**Affiliations:** Rheumatology Department, The Dudley Group, Dudley, United Kingdom; Rheumatology Department, The Dudley Group, Dudley, United Kingdom

## Abstract

**Introduction:**

Takayasu’s arteritis (TA) is a chronic granulomatous large-vessel vasculitis affecting the aorta and its major branches, predominantly in young women. Early diagnosis is challenging because constitutional symptoms may be absent and vascular involvement can initially mimic non-inflammatory vascular disease. Carotid artery stenosis and occlusive disease are recognised manifestations of TA; however, presentation as apparent isolated carotid artery dissection is uncommon and may delay diagnosis. Antiphospholipid antibodies have occasionally been reported in TA and may further complicate diagnostic interpretation in young patients presenting with cerebrovascular events. We present a case illustrating these diagnostic challenges.

**Case description:**

A woman in her 20s presented with transient ischaemic attack symptoms and severe headaches. Neuroimaging demonstrated left internal carotid artery occlusion with small cortical infarcts in the left cerebral hemisphere. Vascular imaging was initially interpreted as spontaneous carotid artery dissection. Serial imaging confirmed persistent occlusion with collateral cerebral circulation via the circle of Willis.

Her medical history included migraine with aura, trigeminal neuralgia, obstructive sleep apnoea, anxiety, depression and vitamin B12 deficiency. A hereditary connective tissue dissection panel was negative. Autoimmune investigations showed negative ANA, ENA, rheumatoid factor, anticardiolipin antibodies and anti-β2 glycoprotein I antibodies, while lupus anticoagulant remained persistently positive on two occasions more than 12 weeks apart.

Over the following two years, she developed progressive fatigue, inflammatory-type arthralgia affecting the hands, ankles and lower back, together with reduced exercise tolerance. Examination demonstrated generalised joint hypermobility without clinical synovitis.

Cross-sectional imaging performed for abdominal pain demonstrated mesenteric and retroperitoneal lymphadenopathy. Repeat contrast-enhanced imaging identified circumferential thickening of the left common carotid artery. Doppler ultrasonography confirmed heterogeneous inflammatory wall thickening with near-complete occlusion extending into the internal carotid artery. A significant inter-limb blood pressure discrepancy further supported large-vessel vasculitis.

A diagnosis of Takayasu’s arteritis presenting as carotid vasculitis mimicking arterial dissection was established. She commenced high-dose prednisolone followed by methotrexate, hydroxychloroquine and antiplatelet therapy, with subsequent improvement in fatigue and functional symptoms.

**Discussion:**

This case highlights an important diagnostic pitfall in large-vessel vasculitis, where Takayasu’s arteritis may initially present as apparent isolated carotid artery dissection with cerebral ischaemia.

Although carotid artery involvement is well recognised in Takayasu’s arteritis, presentation with imaging initially interpreted as spontaneous carotid artery dissection is distinctly uncommon and may delay recognition of the underlying inflammatory vasculopathy. Progressive vascular wall thickening on serial imaging, Doppler evidence of inflammatory arterial disease and a significant inter-limb blood pressure discrepancy ultimately established the diagnosis.

Persistent lupus anticoagulant positivity added further diagnostic complexity. Antiphospholipid antibodies have been described in Takayasu’s arteritis, although their prevalence and clinical significance remain uncertain. This case illustrates that persistent lupus anticoagulant positivity should not prematurely anchor the diagnosis to antiphospholipid syndrome when the clinical course suggests an alternative inflammatory vasculopathy. Careful longitudinal reassessment and repeat vascular imaging were essential in reaching the correct diagnosis.

Recognition of the inflammatory aetiology fundamentally altered management, allowing prompt immunosuppressive therapy aimed at preventing further vascular injury and disease progression.

This case emphasises the importance of maintaining a broad differential diagnosis in young patients presenting with carotid occlusion, dissection-like vascular imaging or cerebrovascular events, particularly when vascular abnormalities evolve over time or systemic inflammatory features emerge.

**Key learning points:**

1. Takayasu’s arteritis may present as an apparent isolated carotid artery dissection or carotid occlusion before classical systemic features develop.

2. Arterial dissection is an uncommon manifestation of Takayasu’s arteritis and may reflect inflammatory vessel wall injury.

3. Persistent lupus anticoagulant positivity should not preclude consideration of Takayasu’s arteritis when the clinical picture is atypical.

4. Serial vascular imaging and longitudinal clinical reassessment are essential in atypical cerebrovascular presentations.

